# Two Monoclonal Antibodies Recognising aa 634-668 and aa 1026-1055 of NogoA Enhance Axon Extension and Branching in Cultured Neurons

**DOI:** 10.1371/journal.pone.0088554

**Published:** 2014-02-12

**Authors:** Bin Deng, Fei Gao, Fang-Fang Liu, Xiang-Hui Zhao, Cai-Yong Yu, Gong Ju, Li-Xian Xu, Jian Wang

**Affiliations:** 1 Institute of Neurosciences, the Fourth Military Medical University, Xi'an, China; 2 Department of Anesthesiology, Stomatological College, Fourth Military Medical University, Xi'an, China; 3 Department of Clinical Laboratory, No. 174 Hospital of People's Liberation Army, Xiamen, China; Agency for Science, Technology and Research - Singapore Immunology Network, Singapore

## Abstract

In a previous study, we generated two monoclonal antibodies (mAbs) in mice, aNogoA-N and aNogo-66 mAb, which were raised against recombinant N-terminal fragments of rat NogoA and Nogo-66, respectively. When compared with the commercial rabbit anti-rat NogoA polyclonal antibody (pAb), which can specifically recognise NogoA, the two mAbs were also specific for the NogoA antigen in immunofluorescence histochemical (IHC) staining and Western blot (WB) analysis. Serial truncations of NogoA covering the N-terminal region of NogoA (aa 570–691) and Nogo-66 (aa 1026–1091) were expressed in *E. coli*. The epitopes recognised by aNogoA-N and aNogo-66 are located in the aa 634–668 and aa 1026–1055 regions of NogoA, respectively. Both mAbs remarkably enhanced the axon growth and branching of cultured hippocampal neurons *in vitro*. These results suggest that the antibodies that bind to aa 634–668 and aa 1026–1055 of NogoA may have stimulatory effects on axon growth and branching. Additionally, the two mAbs that we generated are specific for NogoA and significantly block NogoA function. In conclusion, two sites in NogoA located within aa 634–668 and aa 1026–1055 are recognised by our two antibodies and are novel and potentially promising targets for repair after central nervous system (CNS) injury.

## Introduction

After injury, the central nervous system (CNS) of adult mammals is limited in its ability to recover because of the inability of damaged axons to reconnect and regain their physiological structure and function [1]. Factors that influence axon regeneration include neural cell-autonomous activity, glial scarring, local inflammation, and inhibition factors [2], [3]. In the past two decades, several CNS myelin-derived axon growth inhibitory factors have been found, including NogoA, myelin-associated glycoprotein (MAG), and oligodendrocyte myelin glycoprotein (OMgp) [4], [5], [6]. These proteins have been the subject of great research interest and are highly clinical relevant. NogoA plays an important role in recovery from spinal cord injury, oligodendrocyte differentiation and myelination [7], [8], [9], and the development of the CNS [10], [11]. A recent study reported that a region of NogoA (aa 290–562) attenuates cerebral ischaemia by inhibiting NADPH oxidase-mediated oxidative damage and neuronal apoptosis [12], indicating that the functional diversity of different fragments of NogoA must be explored.

The function of NogoA has been studied extensively using anti-NogoA antibodies [13], [14]. In a previous study, we developed two anti-NogoA monoclonal antibodies, aNogoA-N and aNogo66 mAb, which were generated in mice using recombinant aa 570–691 and aa 1026–1091 fragments, respectively, from NogoA [15]. In the present study, we analysed the specificity and affinity of the two mAbs to the NogoA molecule. We also detected the different epitopes in NogoA that could be recognised by the two mAbs. Using *in vitro* experiments, we found that these mAbs against NogoA enhanced axon growth and branch formation.

## Materials and Methods

### Animals

Male Sprague–Dawley rats weighing 200–220 g and Sprague–Dawley rat embryos (E18.5) were obtained from the Experimental Animal Center of the Fourth Military Medical University (Xi'an, China). All experimental procedures were approved by the Ethics Committee for Animal Experimentation of the Fourth Military Medical University. The protocols used in this research project complied with the guidelines for the care and use of laboratory animals of the Fourth Military Medical University. During the experiments, all efforts were made to minimise animal suffering and the number of animals used.

### Antibodies and reagents

Two hybridoma strains for the mouse anti-rat NogoA protein were preserved by the Institute of Neurosciences in the Fourth Military Medical University, and the mouse IgG was purified as described previously [15]. We purchased the following primary antibodies: polyclonal rabbit anti-NogoA antibody (pAb) (Alpha Diagnostic Intl., USA), rabbit anti-MBP mAb, rabbit anti-GFAP mAb (Denmark DAKO, USA), rabbit anti-GST (Sigma, USA), anti-Tau (Abcam, USA), anti-Map2 (Sigma, USA), anti-βIII-tubulin (Anbo, USA), and anti-β-actin (Anbo, USA). The following secondary antibodies were used: (FITC)-labelled goat anti-mouse immunoglobulin (IgG), Alexa-594-labelled goat anti-rabbit IgG (Abcam, USA), and hydrogen peroxidase (HRP)-conjugated goat anti-rabbit and anti-mouse IgG (Jackson Immuno Research Company, USA). Recombinant Rat NogoA/Fc Chimera (aa 544–725) and Recombinant Rat NogoA/Fc Chimera (aa 1026–1090) were purchased from R&D Systems.

### Western blot and IHC staining

The protein extract from the spinal cord tissues of Sprague-Dawley rats was separated by 10% sodium dodecyl sulphate-polyacrylamide gel electrophoresis (SDS-PAGE) and transferred onto Hybond-P PVDF membranes (Amersham Biosciences) using the Trans-Blot SD Semi-Dry Transfer cell (Bio-Rad) following the manufacturer's instructions. One transferred membrane was blocked with 3% skim milk and 3% bovine serum albumin (BSA) in PBS containing 0.1% Tween-20 for 2 h and incubated with the commercial anti-NogoA pAb (1∶500, 1∶5000, 1∶20000), which was used as positive control, and the other two transferred membranes were incubated with aNogo66 mAb and aNogoA-N mAb (1∶500, 1∶5000, 1∶20000) (1 mg/mL stock concentration) at 4°C overnight. The membranes were washed three times with washing buffer (PBS, 0.05% Tween-20) and then incubated with HRP-conjugated goat anti-mouse IgG or HRP-conjugated goat anti-rabbit IgG (1∶5000 dilution in blocking buffer) (Rockland) for 1 h at room temperature. The membranes were washed three times with washing buffer before antibody binding was visualised using enhanced chemiluminescence reagents (Lumiglo™; Cell Signaling).

The method used to test the binding of antibodies to the targeted Nogo-A region was as follows: The NogoA FC-(aa 1026–1090) or NogoA FC-(aa 544–725) protein was separated by 10% sodium dodecyl sulphate-polyacrylamide gel electrophoresis (SDS-PAGE) and transferred onto Hybond-P PVDF. Blots were probed with aNogo66 mAb or aNogoA-N mAb (1∶500) at 4°C overnight and incubated with HRP-conjugated goat anti-mouse IgG (1∶5000) (Rockland) for 1 h at room temperature. The membranes was visualised using an enhanced chemiluminescence reagent (Lumiglo™; Cell Signaling).

To detect growth-associated protein 43 (Gap-43) expression, the cultured primary neurons were harvested on the fifth and seventh days, and the total protein concentration of the cells was analysed using a BCA kit (Sigma, CA, USA). Blots were probed with a mouse monoclonal antibody against Gap-43 (1∶500, Santa Cruz, CA, USA) and β-actin (1∶2000; Anbo, USA). Each blot was incubated for 2 h at room temperature. Then, the blots were incubated with HRP-conjugated goat anti-mouse IgG (1∶5000 dilution in blocking buffer) (Rockland) for 1 h at room temperature. The membranes was visualised using an enhanced chemiluminescence reagent (Lumiglo™; Cell Signaling).

For IHC, adult rats were anesthetised by an intraperitoneal injection of an overdose of sodium phenobarbital (100 mg/kg) and were then perfused intracardially with warm saline followed by 4% paraformaldehyde (PFA) (pH 7.4). After perfusion, a 15-mm-length thoracolumbar segment of the spinal cord was removed and put into 25% sucrose in 0.1 M phosphate buffer for 36 h at 4°C. Serial coronal sections of a 12 µm thickness were prepared using a freezing microtome (Leica, CA1900, Germany). The sections were post-fixed in 4% PFA for 1 h at room temperature. Subsequently, the sections were rinsed with 0.01 M phosphate-buffered saline (PBS) and then blocked with 1% BSA (Sigma, USA) in PBS containing 0.3% Triton X-100 for 1 h at room temperature. The sections were divided into six groups for the different primary antibodies: I, aNogo66 mAb and Anti-NogoA pAb; II, aNogoA-N mAb and Anti-NogoA pAb; III, aNogo66 mAb and anti-MBP mAb; IV, aNogoA-N mAb and anti-MBP mAb; V, aNogo66 mAb and anti-GFAP mAb; VI, aNogoA-N mAb and anti-GFAP mAb. All sections were incubated in primary antibody at 4°C for 24 h. After washing with PBS three times, the secondary antibodies were incubated in a dark environment at room temperature for 2 h. The stained sections were then washed with PBS three times and mounted with glycerol. The sections were observed under an Olympus BX-51 microscope.

### Expression and purification of recombinant proteins

**Figure 1:**
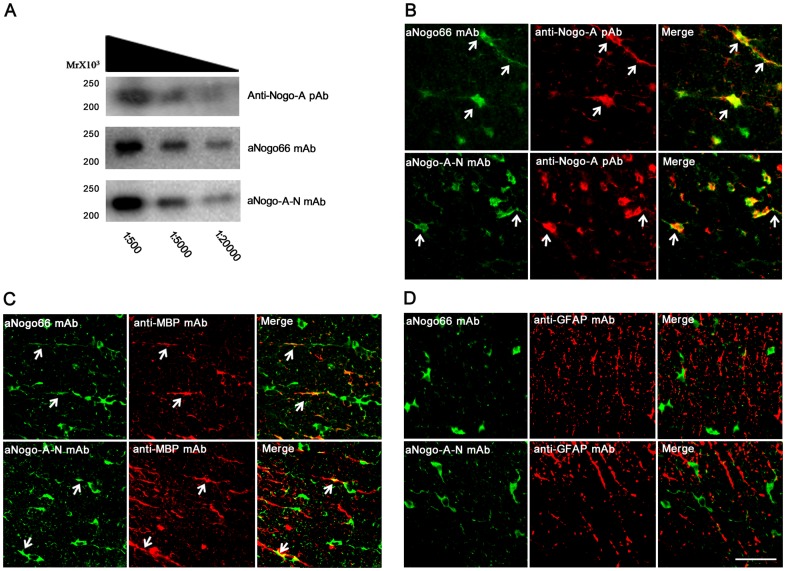
The affinity and specificity of the mAbs to NogoA proteins were determined using Western blot and IHC staining. **A**: The membrane was blotted with different dilutions (1∶500, 1∶5000, 1∶20000) of anti-NogoA pAb, aNogo66 mAb, and aNogoA-N mAb. The corresponding bands were near 200 kDa. **B**: The aNogo66 mAb and aNogoA-N mAb recognised NogoA expressed in oligodendrocytes in the white matter of the spinal cord thoracolumbar coronary segment. Colocalisation (yellow), as indicated by arrowheads, of anti-NogoA pAb (red) with aNogo66 mAb (green) or aNogoA-N mAb (green). **C**: Colocalisation (yellow), as indicated by arrowheads, of anti-MBP mAb (red) with aNogo66 mAb (green) or aNogoA-N mAb (green). **D**: Absence of colocalisation of anti-GFAP mAb (red) with aNogo66 mAb (green) or aNogoA-N mAb (green). Scale bars = 50 µm.

First, we cloned two fragment sequences from the Nogo66 (aa 1026–1091) truncation and four fragment sequences from the NogoA N-terminal (aa 570–691) truncation by reverse transcriptase-polymerase chain reaction (RT-PCR). These fragments were termed ΔNogo-66a (168 bp), ΔNogo-66b (102 bp), ΔNogoA-Na (369 bp), ΔNogoA-Nb (270 bp), ΔNogoA-Nc (171 bp), and ΔNogoA-Nd (99 bp) (Fig. 1A). The following primers with EcoR I and Sal I restriction sites at the 5′ and 3′ ends, respectively, were used:

5′- GGAATTCTATAAGGGCGTGATCCAGG-3′ and 5′-CGTCGACAACTAAGAAAAGCCG CCTCAGTTC-3′; 5′-GGAATTCTCTGAAGTTGCTATATCAGAG-3′ and 5′- CGTCGACAA CTAAGAAAAGCCGCCTCAGTTC-3′; 5′- GGAATTCACAGCACAGCTTTGCCCAT-3′ and 5′-GCGTCGAC GAAATCTGGACTTGGCTCAGTGGAGA-3′; 5′- GAATTCAGCGCTGGT GCTTCTGTAGTG-3′ and 5′-GCGTCGACGAAATCTGGACTTGGCTCAGTG GAGA-3′; 5′-GAATTC CCATATGAAGAAGCCATGAAT-3′ and 5′-GCGTCGAC GAAATC TGGA CTTGGCTCAGTGGAGA-3′; 5′-GAATTC TTTAATGCAGCTGTTCAGGAA-3′ and 5′-GCGTCGAC GAAATCTGGACTTGGCTCAGTGGAGA-3′. Then, the PCR product was subcloned into the EcoR I and Sal I sites of pGEX-4T-1 (Clontech, USA).

After sequencing, all plasmids containing the truncated fragments were transformed into BL21 (DE3) *E. coli* for expression. The recombinants were GST-ΔNogoA-Na, 43 kDa; GST-ΔNogoA-Nb, 38 kDa; GST-ΔNogoA-Nc, 33 kDa; GST-ΔNogoA-Nd, 30 kDa; GST-ΔNogo66a, 33 kDa; GST-ΔNogo66b, 30 kDa. The GST protein is 26 kDa and was expressed from the empty pGEX-4T-1 vector. In this study, the expression of recombinant proteins was under the control of the Tac promoter and was induced by isopropyl-β-D-thiogalactopyranoside (IPTG; Sigma-Aldrich Co., St. Louis, MO). After 2 h of shaking, a final concentration of 0.5 mM IPTG was added into the Luria-broth (LB) medium with ampicillin (100 mg/L), and the medium was further agitated for 3 h [16]. Subsequently, the bacterial cells were pelleted at 12000 g for 6 min, buffer was added, and the cells were boiled for 10 min. SDS-PAGE was performed to analyse the molecular size of the recombinant proteins using Coomassie blue staining and WB analysis. WB was performed briefly as follows. The antibodies were diluted as follows: rabbit anti-GST antibody was diluted 1∶30000 in blocking solution and incubated with the blots for 2 h at room temperature; the secondary antibodies were (HRP)-conjugated goat anti-rabbit IgG (1∶5000) and were incubated with the blots for 1 h at room temperature. Antibody binding was visualised using an enhanced chemiluminescence reagent (Lumiglo TM; Cell Signaling).

The recombinant proteins were purified by affinity chromatography using a pre-charged Ni-NTA Sepharose column (Qiagen Inc., Valencia, CA). The dialysis purity was verified by SDS-PAGE, and the protein concentration was determined by the bicinchoninic acid (BCA) protein quantitation method (Nanjing Jiancheng, China) (Data not shown). Samples were stored at −20°C until use.

### Axon outgrowth and branch formation

Glass coverslips (1 cm) were coated with poly-L-lysine (5 µg/mL), washed three times, and subsequently coated with NogoA FC (aa 1026–1090) or NogoA FC (aa 544–725) (100 pmol, diluted in PBS) for 2 h at 37°C. Unbound NogoA was removed by three washes with PBS. To evaluate the blocking function of the mAbs, glass coverslips were coated with two mAbs (30 µg/ml) for 1 h at 37°C.

Primary hippocampal neurons cultures were prepared as previously described [17]. Briefly, the hippocampal neurons were acquired from SD rat embryos (E18.5). The pups were anesthetised, and 75% ethanol was sprayed on the animals for 5 min. The neurons were isolated and washed with D-Hank's solution three times under sterile conditions. Cells were seeded at a density of 1×10^6^ cells/cm^2^ onto plates and maintained in a humidified incubator (Forma Scientific CO_2_ 3110, Thermo Electron Corporation, USA) at 5% CO_2_ and 37°C. The neurons were cultured in Neurobasal (Gibco, Invitrogen Corp., CA, USA) supplemented with 2% B27 (Gibco, Invitrogen Corp., CA, USA), 1% glutamine (Sigma-Aldrich Corp., St. Louis, MO, USA), and 1% penicillin/streptomycin (Sigma-Aldrich Corp, USA). Half of the medium was changed twice a week. The purity of neurons was determined by immunocytochemistry for βIII-tubulin, and the analysis indicated that 95% of the cells in the cultures were βIII-tubulin (1∶250; Anbo, USA) positive (data not shown).

To observe axon outgrowth, cells were used for immunostaining on the seventh day after culture. The cells were washed after fixation in 4% PFA and then stained with anti-Map2 (1∶1000) and anti-Tau (1∶1000). Measurement of axon length was performed as follows. Five randomly chosen fields of view from the coverslips were photographed using a phase-contrast Olympus IMT2 microscope and an F-View camera at 20× magnification. The axon length per Tau-stained neuron was then measured from these photographs. Statistical significance was assessed using Student's t test. The measurements of the total number of Tau-positive neurons from three independent experiments were analysed by the t-test.

To observe branch formation, the number of axon branch points of per neuron and the distance at which the axon sent out its first branches from the cell body were measured using a phase-contrast Olympus IMT2 microscope and an F-View camera at 40× after examining five randomly chosen fields of view from the coverslips. Statistical significance was assessed using Student's t test. The measurements from three independent experiments were analysed by the t test.

### Statistical analysis

SPSS 13.0 software for Windows (SPSS Inc., Chicago, IL) was used for the statistical analyses. All the data are presented as the means ± SEM. Differences between the groups were assessed by one-way ANOVA followed by the LSD-t test. P values <0.05 were considered to be significant.

## Results

### The two mAbs we generated specifically recognised NogoA protein

The specificity and the affinity of the two mAbs were tested by WB (Fig. 1A). The two mAbs and the commercial rabbit anti-NogoA polyclonal antibody bound to NogoA from spinal cord tissue, with corresponding bands at 200 kDa. Additionally, aNogo66 mAb and aNogoA-N mAb, at different concentrations, strongly bound the NogoA molecule at 200 kDa, indicating that the two mAbs specifically recognise NogoA and have a good affinity for NogoA.

NogoA localises to the membrane surface, the cytoplasm, and processes in oligodendrocytes. First, IHC staining was used to determine the reactivity and specificity of the mAbs in spinal cord tissue from rats. The aNogo-N mAb and aNogo-66 mAbs were double-labelled with commercial rabbit anti-NogoA polyclonal antibody (pAb) in spinal cord sections (Fig. 1B). Furthermore, the two mAbs colocalised with MBP, which is a positive marker for oligodendrocyte neurites and myelin (Fig. 1C). However, the two mAbs did not double-stain with GFAP (Fig. 1D). These results suggest that the two mAbs specifically recognise NogoA.

### The epitopes recognised by the aNogo-N and aNogo-66 mAbs are located within aa 634-668 and aa 1026-1055 of NogoA, respectively

**Figure 2:**
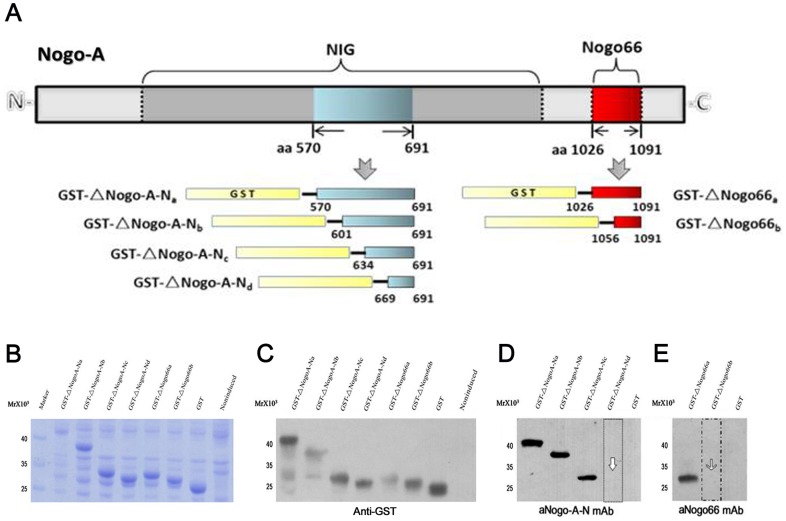
Identification of the epitopes recognised by the two mAbs by Western blot. **A**: Several peptide epitopes from NogoA were produced. A library of six Nogo deletion constructs was made, and GST-tagged recombinant proteins were designed. **B**: Lysates of induced BL21 bacteria with recombinant plasmids were analysed using Coomassie brilliant blue staining (lanes from left to right: lysates of induced BL21 bacteria with recombinant plasmids GST-ΔNogoA-Na, GST-ΔNogoA-Nb, GST-ΔNogoA-Nc, GST-ΔNogoA-Nd, GST-ΔNogo66a, and GST-ΔNogo66b; lane 7: lysate of induced BL21 bacteria with empty pGEX-4T-1 vector; lane 8: lysates of non-induced BL21 bacteria alone). **C**: Recombinant proteins identified by WB. Anti-GST antibody was used to detect the fragments (lanes from left to right: the recombinant proteins GST-ΔNogoA-Na, GST-ΔNogoA-Nb, GST-ΔNogoA-Nc, GST-ΔNogoA-Nd, GST-ΔNogo66a, and GST-ΔNogo66b; lane 7: GST protein; lane 8: non-induced). **D**: The epitope recognised by the aNogoA-N mAb (lanes from left to right: the recombinant proteins GST-ΔNogoA-Na, GST-ΔNogoA-Nb, GST-ΔNogoA-Nc, GST-ΔNogoA-Nd and GST protein). **E**: The epitope recognised by the aNogo66 mAb (lanes from left to right: the recombinant proteins GST-ΔNogo66a and GST-ΔNogo66b and GST protein).

To identify the location of the epitopes in NogoA that are recognised by the aNogo-N and aNogo-66 mAbs, six truncations of NogoA were produced as recombinant GST-fused peptides (Fig. 2A). All the recombinants displayed their predicted molecular weights after SDS-PAGE and Coomassie blue staining (Fig. 2B). Subsequently, we used an anti-GST antibody to detect these recombinant proteins by WB. Recombinant proteins in all lanes were recognised specifically by the anti-GST antibody except for lane 8, where the protein was not expressed without IPTG induction (Fig. 2C). Next, the GST-tagged fusion proteins were purified by Ni-NTA agarose affinity chromatography. After the bound proteins were eluted, the purity of the final products in the portion of the eluate was approximately 95% (data not shown).

To detect the location of the epitopes in NogoA that are recognised by aNogo-N mAb and aNogo-66 mAb, we screened the recombinants by WB assay. The aNogoA-N mAb recognised the GST-ΔNogoA-Na, GST-ΔNogoA-Nb, and GST-ΔNogoA-Nc peptides but not the GST-ΔNogoA-Nd peptide (Fig. 2D). This result suggested that the epitope recognised by aNogoA-N mAb is located between aa 634 and 668 of NogoA (Table S1). Using the same method, the aNogo66 mAb only bound the GST-ΔNogo66a peptide but did not recognise the GST-ΔNogo66b peptide (Fig. 2E). This result suggested that the epitope recognised by aNogoA-N mAb is located between aa 1026 and 1055 of NogoA (Table S1).

### Both mAbs prevented the inhibition of NogoA fragments on axonal extension and branching in vitro

**Figure 3:**
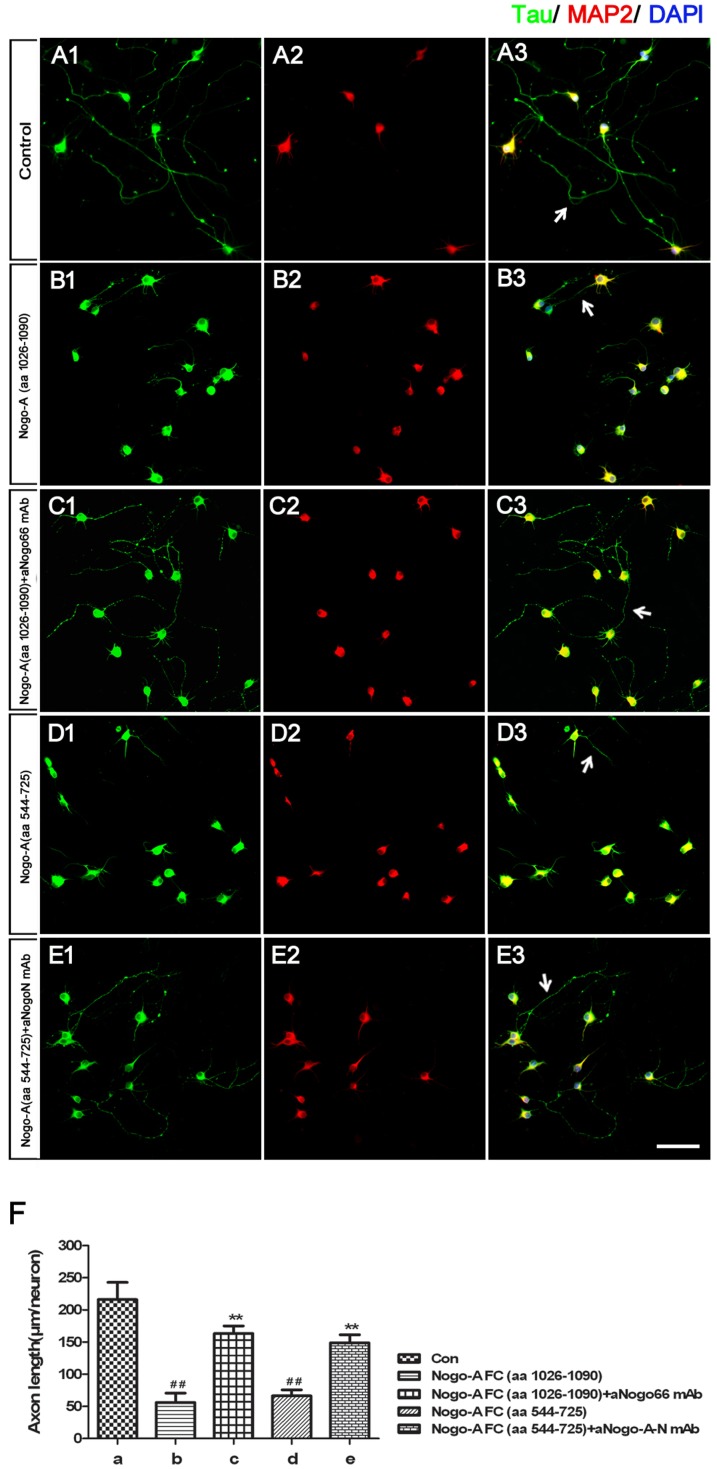
aNogo66 mAb and aNogoA-N mAb enhanced the axon growth of hippocampal neurons by blocking NogoA inhibition. **A**: Dissociated rat E18.5 hippocampal neurons were cultured on the control substrate PLL (control group). **B, D**: Hippocampal neurons cultured on 100 pmol NogoA FC (aa 1026–1090) or NogoA FC (aa 544–725) exhibited strongly inhibited axon growth. **C, E**: Hippocampal neurons were cultured on NogoA FC (aa 1026–1090) and treated with aNogo66 mAb, or neurons were cultured on NogoA FC (aa 544–725) and treated with aNogoA-N mAb, to assess the contribution of the two mAbs on inhibition. **F**: Statistical analysis of hippocampal neuron axon growth in each group is expressed as the mean ± SEM of each group in each separate experiment (^##^ *P*<0.01, b group or d group vs. a group; ^**^ *P*<0.01, c group vs. b group and e group vs. d group. n = 6 wells per condition; scale bars  = 100 µm).

**Figure 4:**
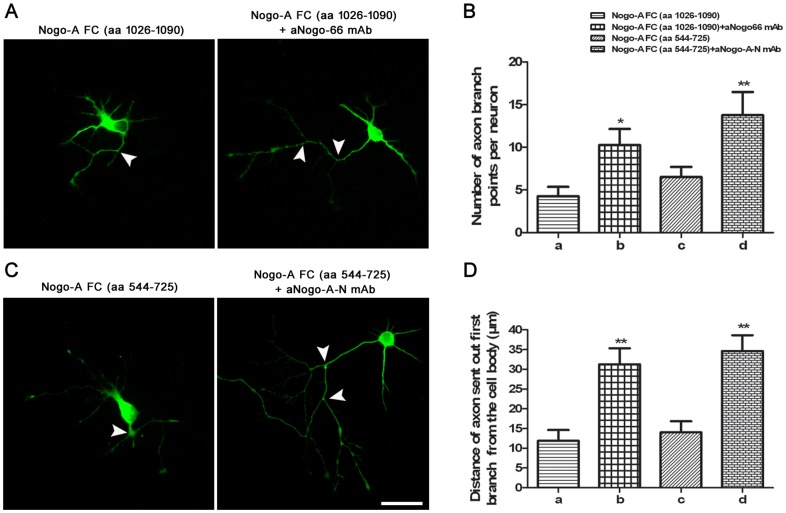
The aNogo66 mAb and aNogoA-N mAb enhanced branch formation and suppressed myelin inhibition. **A, C**: The function of aNogo66 mAb or aNogoA-N mAb on branch formation against NogoA was assessed by immunofluorescence. The arrowheads indicate the axon branch points (n = 6 wells per condition; scale bars  = 50 µm). **B**: For statistical analysis, the number of axon branch points per neuron is represented as the mean ± SEM from one representative experiment (^*^ *P*<0.05, b group vs. a group; ^**^ *P*<0.01, d group vs. c group). **D**: For statistical analysis, the distance that axons sent out their first branches from the cell body was expressed as the mean ± SEM from one representative experiment (^**^ *P*<0.01, b group vs. a group; d group vs. c group).

Hippocampal neurons contain a long axon under normal culture conditions *in vitro* (Fig. 3A). The axon extension of cultured hippocampal neurons was significantly inhibited by NogoA FC- (aa 1026–1090) or NogoA-FC (aa 544–725) treatment *in vitro* (*P*<0.01) (Fig. 3B, D, E). Compared with the control group, axon extension was almost completely rescued by adding aNogoA-N mAb to the NogoA FC- (aa 544–725) treated group or by adding aNogo66 mAb to the NogoA FC- (aa 1026–1090) treated group (Fig. 3C, D, E). We next examined the function of the mAbs on axonal branching in cultured neurons *in vitro*. The number of branching points in the mAb groups was greater than either of the NogoA FC groups (*P*<0.05) (Fig. 4A, B, C). The axons of cells in the mAb groups sent out their first branches farther away from the cell body than did axons from cells in either of the NogoA FC groups (*P*<0.01) (Fig. 4 A, C, D). These results showed that the two mAbs we generated can block NogoA fragments that inhibit axon extension and branching *in vitro*.

### Two mAbs reduced the inhibition exerted by the targeted Nogo-A region on axon outgrowth and branching

**Figure 5:**
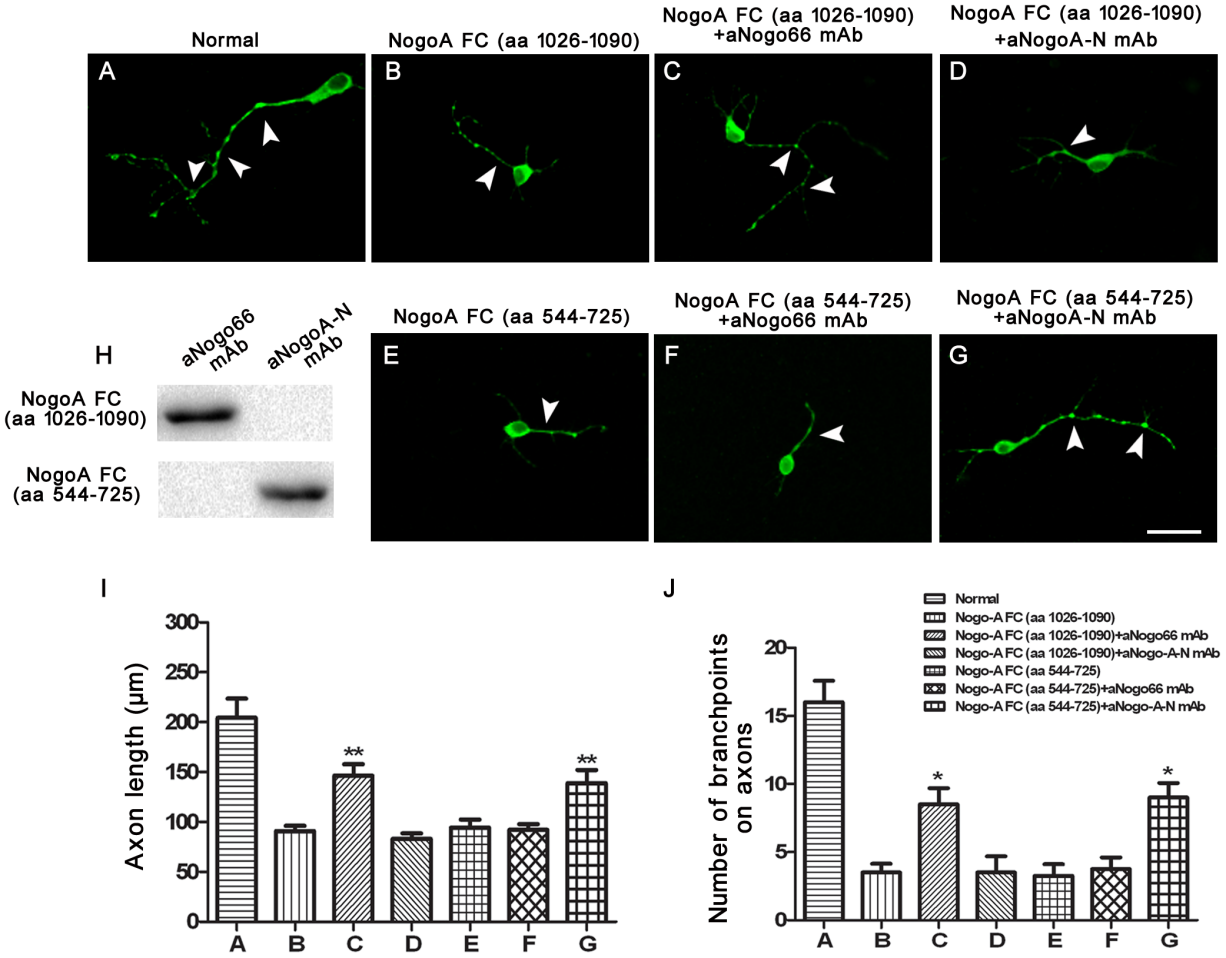
aNogo66 mAb and aNogoA-N mAb reduce the inhibition exerted by the targeted Nogo-A region on axon outgrowth and branching. A: Dissociated rat E18.5 hippocampal neurons were cultured on the control substrate PLL (Normal group). B, C, D: Hippocampal neurons cultured on 100 pmol NogoA FC (aa 1026–1090); E, F, G: Hippocampal neurons cultured on 100 pmol NogoA FC (aa 544–725); C, F: Treated with aNogo66 mAb; D, G: treated with aNogoA-N mAb. I: Statistical analysis of the axon growth is expressed as the mean ± SEM of each group in each separate experiment. J: Statistical analysis of the number of axonal branch points is expressed as the mean ± SEM of each group in each separate experiment (^**^ *P*<0.01, C group vs. B or D group; ^**^ *P*<0.01, G group vs. E or F group; ^*^ *P*<0.05, C group vs. B or D group; ^*^ *P*<0.05, G group vs. E or F group; scale bars  = 100 µm)

WB analysis showed that the aNogo66 mAb could bind to NogoA FC- (aa 1026–1090) but did not recognise NogoA FC-(aa 544–725). The aNogoA-N mAb recognised NogoA FC- (aa 544–725) but not the NogoA FC-(aa 1026–1090) (Fig. 5 H).

The axon extensions were almost completely rescued by adding the aNogoA-N mAb to the NogoA FC- (aa 544–725) or by adding the aNogo66 mAb to the NogoA FC- (aa 1026–1090) (Fig. 5 C/G). However, aNogoA-N could not rescue axon outgrowth on Nogo-A FC-(aa 1026–1090), and aNogo66 mAb could not rescue axon outgrowth on NogoA FC- (aa 544–725) (Fig. 5 D/F). The number of branching points obtained by adding the aNogoA-N mAb to the NogoA FC- (aa 544–725) treated group or adding the aNogo66 mAb to the NogoA FC- (aa 1026-1090) treated group was more than that in the Nogo-A FC-(aa 1026–1090) or NogoA FC- (aa 544–725) group (Fig. 5 C/G). However, no obvious effect was produced by adding the aNogoA-N mAb to the NogoA FC- (aa 1026–1090) or adding the aNogo66 mAb to the NogoA FC- (aa 544–725) (Fig. 5 D/F). These results showed that the two mAbs reduced the inhibition exerted by the targeted Nogo-A region on axon outgrowth and branching.

### The neurons treated with the two mAbs upregulated GAP-43 in vitro

**Figure 6:**
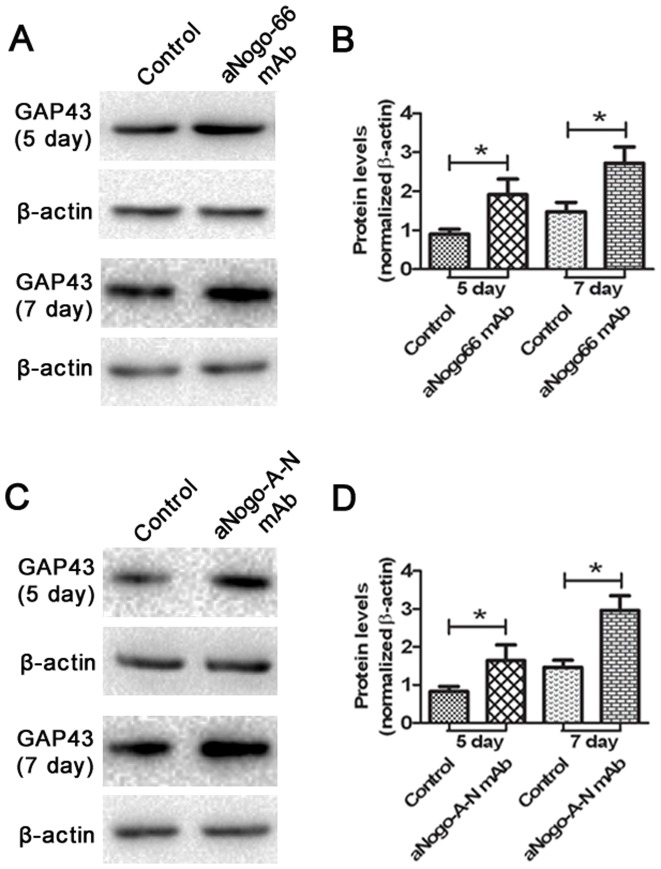
Western blot analysis of the levels of GAP43 expression on the fifth and seventh days after primary hippocampal neuron culture *in vitro*. (n = 6 wells per condition). A: Hippocampal neurons were grown on 100 pmol NogoA FC (aa 1026–1090) substrate for the control group; hippocampal neurons were grown on NogoA FC (aa 1026–1090) and treated with aNogo66 mAb for the Nogo66 mAb group; B: Data are expressed as the level of the GAP43-positive band in the control group and the Nogo66 mAb group on the fifth day or seventh day. ^*^ *P*<0.05. C: Hippocampal neurons grown on the NogoA FC (aa 544–725) substrate were the control group; hippocampal neurons grown on NogoA FC (aa 544–725) and treated with aNogoA-N mAb were the aNogoA-N mAb group; D: Data are expressed as the level of GAP43-positive substance in the control group and the aNogoA-N mAb group on the fifth day or seventh day. ^*^ *P*<0.05.

GAP-43 plays a critical role in axonal extension and branching [1]. We next assayed the expression level of GAP-43 in cultured neurons after mAb treatment *in vitro* (Fig. 6). The level of GAP-43 was significantly higher in both the aNogo-66 mAb and aNogoA-N mAb treatment groups than the NogoA FC (aa 1026–1090) and NogoA FC (aa 544–725) groups. These results implied that NogoA may inhibit axonal extension and branching via the downregulation of GAP-43.

## Discussion

Rat NogoA is a member of the reticulon family of transmembrane proteins. The full-length rat NogoA is 1163 aa in length and contains a 989 aa N-terminus, a 21 aa transmembrane segment, a 94 aa connecting “loop”, a second 21 aa transmembrane segment, and a 38 aa C-terminus [18]. The potent inhibitory activities of the different NogoA regions have been studied previously. Three areas are of particular interest. One such area is Nogo-66 (aa 1026–1091) in the C-terminal region of NogoA, which is reported to bind to the GPI-linked Nogo receptor/p75 complex on axons and induce growth cone collapse [19], [20]. Two other regions in the N-terminus have also been discovered to have bioactivity. Amino acids 59–172 are reported to block fibroblast spreading, whereas Nogo-Δ20 (aa 544–725) exerts strong inhibitory effects on growing neurites and growth cones *in vitro* and, unlike Nogo-66, also on the migration of non-neuronal cells such as fibroblasts [21], [22], [23].

Furthermore, an anti-serum (AS 472) against aa 623-640 in the NogoA-specific region neutralises the inhibitory activity of CNS myelin *in vitro* and induces the sprouting of adult rat Purkinje axons *in vivo* [21]. Using function-blocking NogoA-specific antibodies to block NogoA or a soluble Nogo-66-binding fusion protein comprising the domains of NgR1, using antagonistic peptides, or blocking Rho-A and its downstream target ROCK can improve regeneration [1].

In our previous study, we developed two different monoclonal antibodies, aNogo66 and aNogoA-N mAbs, which were produced against aa 570–691 and aa 1026–1091 of the rat NogoA protein [15]. However, there were many basic research and clinical application questions to be explored. For example, the epitopes of the mAbs needed to be identified, and the blocking function of the mAbs needed to be elucidated.

In the present study, we found that the two mAbs spefocifically recognise NogoA in tissues. The aNogo66 mAb recognises an epitope within aa 1026–1055. Interestingly, another region of Nogo66 (aa 1055–1099 of NogoA), on the surface of oligodendrocytes, can be recognised by the antibody AS 922, which can block the neurite growth inhibitory activity of NogoA [21]. Therefore, aa 1026–1055 may be a new functional region in NogoA, and the aNogo66 mAb may have valuable applications in the future. The aNogoA-N mAb recognises an epitope within aa 634-668, which is different from the synthetic peptide corresponding to the rat sequence (aa 623–640) for mAb 11C7, which enhances axon growth and fibroblast spreading [23]. Other reports have shown that amino-NogoA antagonises reactive oxygen species generation and protects immature primary cortical neurons from oxidative toxicity [24]. These findings suggested that aa 634–668 may be another new functional region of NogoA.

NogoA is highly expressed in outgrowing neurons *in vivo* [25], [26], including in growth cones. In the CNS, adult mice lacking NogoA showed an upregulation of cytoskeletal and growth-related mRNAs and proteins in the spinal cord and cortex [27]. Furthermore, the addition of function-blocking NogoA-specific antibodies induces both the upregulation of growth-specific proteins and pronounced neurite sprouting in hippocampal neurons [28]. Notably, during these processes, GAP-43 plays an important role coincident with myelin formation [29], [30], [31], [32]. A remarkable congruence has been found in the effects of different blocking agents for NogoA signalling, including antibodies, receptor bodies, and small molecule blockers, in a number of regeneration and plasticity paradigms [1], [33], [34]. An antibody that blocks NogoA function has reached the clinical trial stage as a novel treatment for spinal cord injury [14], [35], [36].

NogoA exerts repulsive and neurite growth-inhibitory functions in the CNS of developing and adult animals and can be found in the innermost membrane and in the outer myelin membrane in oligodendrocytes [25], [37]. Additionally, NogoA is highly expressed in outgrowing neurons *in vivo*, including in growth cones and at synapses [37]. Nogo receptor 1 (NgR1) is one part of the functional Nogo receptor complex with proteins such as LINGO1 and the presumed signal transducers p75 and Troy [38]. In addition, NgR1 is expressed on the nerve cell body, in growth cones, and at synapses [19]. *In vivo*, the acute blockade of NgR1 enhanced sprouting, regeneration, and plastic rearrangements of fibre connections after CNS injury in adult rats [39], [40]. Thus, a NogoA antibody can inhibit NogoA–NgR binding. Treatments targeting Nogo signalling resulted in the most consistent and extensive structural and functional recoveries after spinal cord or stroke lesions [40,]41,42]. A clinical study in patients with acute injuries in the spinal cord with a human NogoA antibody is currently underway. Therefore, our research may have important applications for basic to clinical studies of spinal cord or stroke lesions.

In summary, the epitope sequences recognised by the aNogo66 mAb and aNogoA-N mAb were determined, and the specificity and affinity of the mAbs to NogoA were demonstrated. Furthermore, we demonstrated that both mAbs reverse the inhibitory effect of NogoA protein on axon growth and branch formation *in vitro*. Moreover, these findings suggest that the epitope sequences may constitute new functional regions of NogoA that can regulate growth cone collapse and neuronal plasticity. The mAbs may benefit studies of the function of NogoA, particularly studies of target and short-peptide drugs for the treatment of CNS injury.

## Supporting Information

Table S1 **Amino acid sequences of peptide of Rat NogoA recognised by aNogo66 mAb or aNogoA-N mAb, respectively.**
(DOCX)
