# Supplementary material for: Two Monoclonal Antibodies Recognising aa 634-668 and aa 1026-1055 of NogoA Enhance Axon Extension and Branching in Cultured Neurons
Source: PLoS One. 2014 Feb 12;9(2):e88554. doi: 10.1371/journal.pone.0088554 (PMC3922884; doi:10.1371/journal.pone.0088554)
Supplement: Table S1 — Amino acid sequences of peptide of Rat NogoA recognised by aNogo66 mAb or aNogoA-N mAb, respectively. (DOCX) [file pone.0088554.s001.docx]

**Table S1:** Amino acid sequences of peptide of Rat NogoA recognised by aNogo66 mAb or aNogoA-N mAb, respectively.


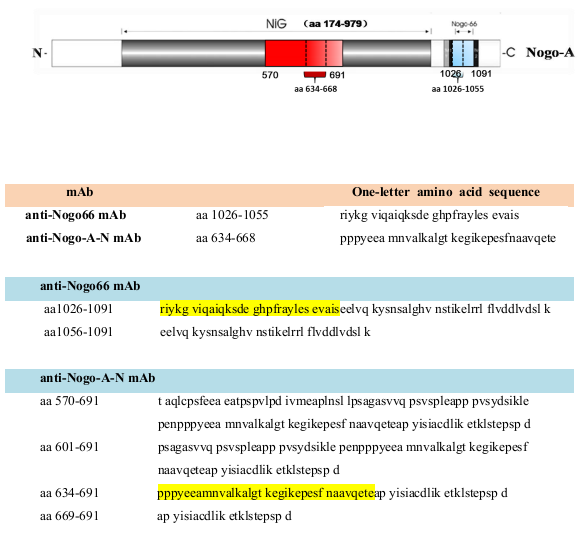


Note: The epitope recognised by aNogoA-N mAb is located between aa 634 and 668 of NogoA and the epitope recognised by aNogoA-N mAb is located between aa 1026 and 1055 of NogoA, as yellow marked.
